# An Electrospun DFO-Loaded Microsphere/SAIB System Orchestrates Angiogenesis–Osteogenesis Coupling via HIF-1α Activation for Vascularized Bone Regeneration

**DOI:** 10.3390/polym17111538

**Published:** 2025-05-31

**Authors:** Xujia Shan, Xiaoyan Yuan, Xiaohong Wu

**Affiliations:** Chongqing Key Laboratory of Oral Diseases, Chongqing Municipal Key Laboratory of Oral Biomedical Engineering of Higher Education, Chongqing Municipal Health Commission Key Laboratory of Oral Biomedical Engineering, College of Stomatology, Chongqing Medical University, Chongqing 401147, China; 2022110481@stu.cqmu.edu.cn (X.S.); 2022120711@stu.cqmu.edu.cn (X.Y.)

**Keywords:** deferoxamine, poly(lactic-co-glycolic acid) microspheres, angiogenesis, osteogenesis, HIF-1α signaling pathway

## Abstract

This study developed electrosprayed deferoxamine (DFO)-loaded poly(lactic-co-glycolic acid) microspheres (DFO-MS) combined with a sucrose acetate isobutyrate (SAIB) depot (DFO-MS@SAIB) for bone-defect repair, targeting the coordinated regulation of angiogenesis and osteogenesis in vascularized bone regeneration—where new blood vessels support functional bone integration. In vitro/in vivo evaluations confirmed its dual pro-angiogenic and pro-osteogenic effects via HIF-1α pathway activation. Background/Objectives: Emerging evidence underscores the indispensability of vascularization in bone-defect repair, a clinical challenge exacerbated by limited intrinsic healing capacity. While autologous grafts and growth-factor-based strategies remain mainstream, their utility is constrained by donor-site morbidity, transient bioactivity, and poor spatiotemporal control over angiogenic–osteogenic coupling. Here, we leveraged DFO, a hypoxia-mimetic HIF-1α stabilizer with angiogenic potential, to engineer an injectable DFO-MS@SAIB depot. This system was designed to achieve sustained DFO release, thereby synchronizing vascular network formation with mineralized tissue regeneration in critical-sized defects. Methods: DFO-MS were fabricated via electrospraying and combined with SAIB (DFO-MS@S) to form an injectable sustained-release depot. Their physicochemical properties, including morphology, encapsulation efficiency, degradation, release kinetics, and rheology, were systematically characterized. In vitro, the angiogenic capacity of HUVECs co-cultured with DFO-MS was evaluated; conditioned HUVECs were then co-cultured with BMSCs to assess the BMSCs’ cytocompatibility and osteogenic differentiation. In vivo bone regeneration in a rat calvarial defect model was evaluated using micro-CT, histology, and immunohistochemistry. Results: The DFO-MS@SAIB system achieved sustained DFO release, stimulating HUVEC proliferation, migration, and tubulogenesis. In a Transwell co-culture model, pretreated HUVECs promoted BMSC migration and osteogenic differentiation via paracrine signaling involving endothelial-secreted factors (e.g., VEGF). HIF-1α pathway activation upregulated osteogenic markers (ALP, Col1a1, OCN), while in vivo experiments demonstrated enhanced vascularized bone regeneration, with significantly increased bone volume/total volume (BV/TV) and new bone area compared with controls. Conclusion: The DFO-MS@SAIB system promotes bone regeneration via sustained deferoxamine release and HIF-1α-mediated signaling. Its angiogenesis–osteogenesis coupling effect facilitates vascularized bone regeneration, thereby offering a translatable strategy for critical-sized bone-defect repair.

## 1. Introduction

Bone defects resulting from trauma, tumor resection, or congenital disorders represent a significant clinical challenge due to the limited self-healing capacity of bone tissue [1]. Vascularized bone regeneration is defined as the biological process wherein new blood vessel formation actively supports the integration, mineralization, and functional maturation of newly formed bone tissue [2,3,4,5]. Recent studies have demonstrated that vascularization is indispensable for bone-defect repair [6,7]. The spatial–temporal regulation of neovascularization, particularly endothelial-cell-mediated angiocrine signaling and hemodynamic nutrient supply, directly orchestrates osteoprogenitor recruitment and mineralization processes [8,9,10]. Autologous bone grafting, the current gold standard for treating such defects, is constrained by donor-site morbidity, which affects approximately 30% of patients, and the limited availability of viable graft material [11,12]. Synthetic scaffolds, such as hydroxyapatite or calcium phosphate-based materials, offer an alternative but often fail to integrate fully with host tissues due to insufficient angiogenesis and mechanical mismatch, leading to suboptimal repair outcomes [13,14]. Growth factors, including bone morphogenetic protein-2 (BMP-2) and vascular endothelial growth factor (VEGF), have demonstrated potential in enhancing bone regeneration by stimulating osteogenic and angiogenic pathways [15,16]. However, their clinical application is hampered by short half-lives, high production costs, and risks of ectopic ossification when administered at supraphysiological doses [17,18,19,20,21]. These limitations underscore the need for innovative strategies to achieve sustained and localized delivery of therapeutic agents that can effectively promote vascularized bone regeneration.

Hypoxia-inducible factor-1α (HIF-1α), a master regulator of cellular adaptation to hypoxia, has emerged as a pivotal mediator that couples angiogenesis and osteogenesis [22]. Under normoxic conditions, HIF-1α undergoes hydroxylation by prolyl hydroxylase domain (PHD) enzymes, leading to proteasomal degradation via the von Hippel–Lindau (VHL) E3 ubiquitin ligase complex [23]. Deferoxamine (DFO), an FDA-approved iron chelator, stabilizes HIF-1α by competitively binding Fe^2+^ ions required for PHD activity, thereby upregulating downstream targets such as VEGF and BMP-2 [24]. In a rat femoral-defect model, local DFO administration enhanced callus formation compared with controls [25]. Despite its therapeutic potential, systemic DFO delivery risks severe adverse effects, including auditory toxicity and retinal degeneration, while bolus injections fail to sustain therapeutic concentrations beyond 24 h [24]. Consequently, developing biodegradable carriers capable of maintaining DFO within the therapeutic window is critical to achieving bone regeneration [26]. Despite the known pro-angiogenic and osteogenic effects of DFO, its clinical efficacy in bone repair is limited by the lack of a delivery system that balances sustained release and biocompatibility. Existing carriers often suffer from burst release, poor drug-loading capacity for hydrophobic agents, or mismatches between degradation rates and bone repair timelines. Sucrose acetate isobutyrate (SAIB), with its hydrophobic nature and gradual erosion, may overcome these limitations for DFO delivery.

Poly(lactic-co-glycolic acid) (PLGA), a biocompatible and tunable polymer, has been widely employed for sustained drug delivery due to its predictable degradation kinetics (1–6 months) and FDA approval status [27]. Conventional emulsion-based PLGA microspheres, however, suffer from low drug-loading efficiency and significant burst release. Electrospraying technology, which utilizes high-voltage electrostatic forces to generate monodisperse droplets, overcomes these limitations by achieving a relatively high encapsulation efficiency and minimizing organic solvent residues [28]. Our previous studies have shown that the electrospun naringin-loaded microsphere/SAIB system (Ng-m-SAIB) prepared by our research group showed good sustained release performance and certain osteogenic effects on a rat skull defect [29]. However, the bone healing cascade critically depends on early vascularization to establish oxygen and nutrient supply [30,31,32], as failure to construct this vascular network entraps repair processes in a self-perpetuating cycle of avascular necrosis and failed osteogenesis [30,33]. To address this bottleneck, we strategically employed DFO-loaded PLGA electrosprayed microspheres (DFO-MS). While DFO’s vascular benefits are recognized [5,34], its direct osteogenic mechanisms remain elusive, and the therapeutic potential of microsphere-encapsulated DFO in orchestrating coupled angio-osteogenesis has never been systematically investigated. This knowledge gap underscores the necessity of our study to decode DFO-MS’s dual functionality and optimize their spatiotemporal delivery via SAIB depots.

**Scheme 1:**
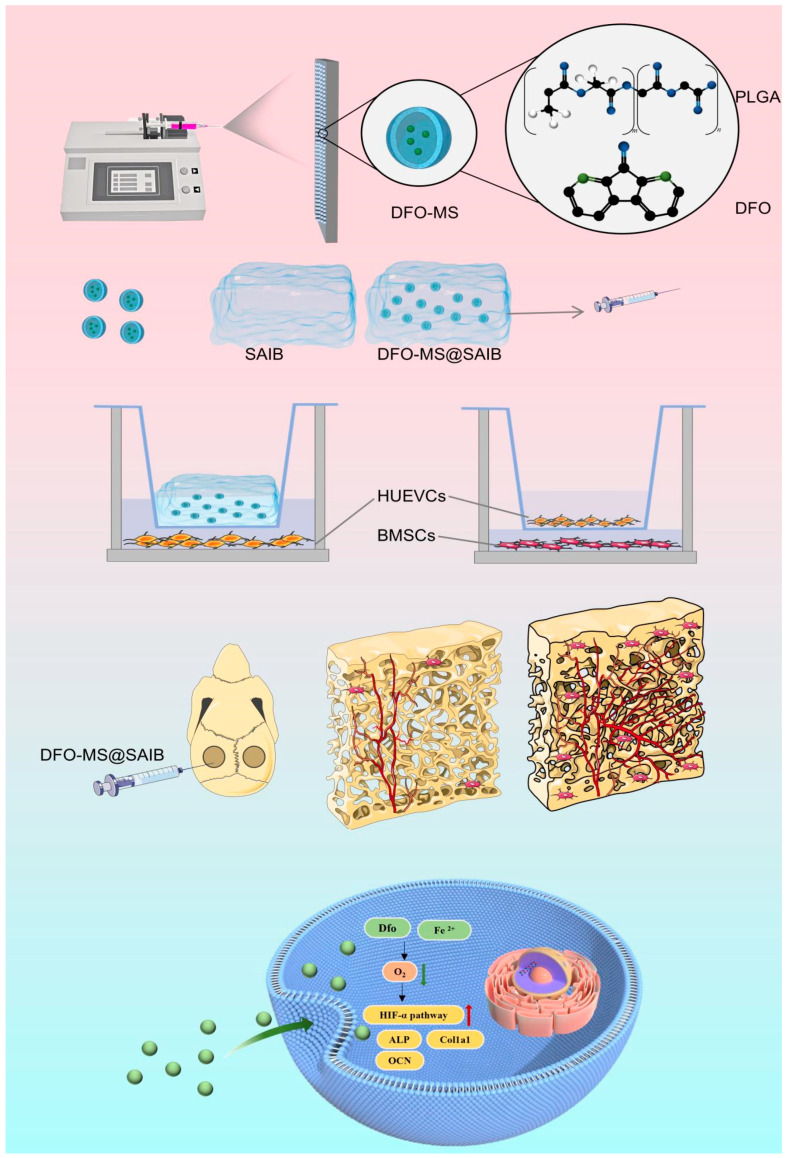
Electrospinning fabricates DFO-loaded PLGA microspheres (DFO-MS), which are then combined with SAIB into an injectable system. It promotes vascularized bone regeneration via the HIF-1α pathway, verified in vitro (HUVECs/BMSCs) and in vivo (rat calvarial defect), achieving angiogenesis—osteogenesis coupling.

This study aims to develop electrosprayed DFO microspheres with optimized physicochemical properties for minimally invasive implantation and to validate the therapeutic efficacy of DFO-M-SAIB microspheres in a critical-sized bone-defect model, bridging material design with in vivo regeneration (Scheme 1). This study provides a translatable strategy for critical-sized defect repair, with potential extensions to other hypoxia-targeted therapies and clinical scalability through minimally invasive percutaneous injection, potentially reducing surgical morbidity and healthcare costs.

## 2. Materials and Methods

### 2.1. Preparation of DFO-MS and DFO-MS@SAIB

The electrosprayed drug-loaded microspheres were fabricated through the following procedure: PLGA (6% *w*/*v* relative to chloroform, lactide/glycolide = 75:25, -COOH end group, Mw = 50,000 Da; Sigma, Saint Louis, MO, USA; purity ≥99%), PEG (polyethylene glycol; 0.3% *w*/*w* relative to PLGA; Sigma; purity ≥99%), and DFO (5–20% *w*/*w* relative to PLGA; Sigma; purity ≥98%) were precisely weighed and dissolved in chloroform under continuous magnetic stirring for 2 h to obtain a homogeneous electrospinning solution. The fully dissolved solution was then loaded into a 5 mL disposable syringe equipped with a 22G stainless steel needle. DFO-loaded microspheres (DFO-MS) were generated using an electrostatic spinning apparatus (Yongkang Leye Technology Development Co., Ltd., Beijing, China) under controlled environmental conditions (temperature: 22–23 °C; relative humidity: 30–31%). The operational parameters were optimized as follows: solution feeding rate maintained at 0.080 mm/min, applied voltage set to 24 kV, and collection distance fixed at 20 cm. The resulting microspheres were collected on aluminum foil and subsequently dried in a constant-temperature oven at 37 °C for 48 h to remove residual solvents. Finally, the dried microspheres were stored at −20 °C in sealed containers for subsequent experimental applications. DFO-MS@SAIB composites with varying drug-loading capacities were prepared by first precisely weighing 6 mg, 8 mg, 10 mg, and 12 mg of DFO-MS. Each aliquot was individually incorporated into 200 μL of SAIB (sucrose acetate isobutyrate; Sigma; purity ≥98%) and homogenized via vortex mixing for 5 min to ensure uniform dispersion of the microspheres within the SAIB matrix. The resulting formulations, designated as DFO-MS@SAIB with distinct microsphere-loading levels, were stored under inert conditions prior to further characterization.

### 2.2. Scanning Electron Microscopy (SEM)

The microsphere-coated samples adhered to aluminum foil (2 cm × 2 cm) were sputter-coated with gold and morphologically characterized using an SEM (ZEISS GeminiSEM 300, Oberkochen, Germany) at an accelerating voltage of 5 kV.

### 2.3. Drug-Encapsulation Efficacy (EE) and Drug-Loading Efficacy (LE)

A 5 mg aliquot of DFO-MS was immersed in a microcentrifuge tube containing 1 mL of phosphate-buffered saline (PBS, PH 7.4) and centrifuged at 14,000 rpm for 10 min. The supernatant was collected and reacted with an excess of iron(III) chloride (purity ≥ 99%). The DFO concentration was quantified at a wavelength of 485 nm using a UV-Vis spectrophotometer (Thermo Fisher Scientific, Waltham, MA, USA). The encapsulation efficiency (EE) was calculated using the following formula:

$$EE\%=\left(1\;-\;\frac{\mathrm{Free}\;\mathrm{drug}}{\mathrm{Amount}\;\mathrm{of}\;\mathrm{drug}\;\mathrm{in}\;\mathrm{the}\;\mathrm{spheres}}\right)\times 100\%$$

A 5 mg aliquot of DFO-MS was immersed in a microcentrifuge tube containing 1 mL of ethanol, subjected to ultrasonic disruption for 10 min, and subsequently centrifuged at 14,000 rpm for 20 min. The supernatant was collected, and the DFO concentration was quantified at a wavelength of 485 nm using a UV-Vis spectrophotometer. The loading efficiency (LE) was calculated using the following formula:

$$LE\%=\frac{\mathrm{Amount}\;\mathrm{of}\;\mathrm{drug}\;\mathrm{in}\;\mathrm{the}\;\mathrm{spheres}}{\mathrm{Weight}\;\mathrm{of}\;\mathrm{the}\;\mathrm{spheres}}\times 100\%$$

### 2.4. Determination of the Contact Angle

The microsphere-coated aluminum foil samples were prepared by collecting microspheres at identical sites on the aluminum foil after electrospinning under the same processing parameters for 30 min. The samples were then cut into 1 cm × 1 cm pieces and mounted horizontally on a contact-angle goniometer (VCA Optima, Billerica, MA, USA; AST Products, Inc., Billerica, MA, USA). A 2 μL droplet of deionized water was automatically dispensed onto the sample surface using a precision syringe, and the static contact angle was measured within 10 s of droplet deposition via sessile drop analysis, with three independent replicates per sample, and the contact angle was measured at 22–23 °C under ambient conditions.

### 2.5. In Vitro Degradation

The degradation kinetics of DFO-MS with varying drug-loading rates (5%, 10%, 15%, 20% *w*/*w* relative to PLGA) were evaluated by incubating pre-weighed samples (10 mg) in phosphate-buffered saline (PBS; pH 7.4, 37 °C) under gentle agitation (70 rpm). At predetermined intervals (0, 7, 14, 21, 28, 35, 42 days), the microspheres were collected by centrifugation (12,000 rpm, 5 min), rinsed with deionized water, lyophilized for 24 h, and reweighed. The degradation mass percentage was calculated using the following formula:

$$Degradation\;mass\;percentage\;=\;\frac{w_{0}-w_{t}}{w_{0}}\;\times \;100\%$$

where W_0_ and W_t_ represent the initial and residual dry weights, respectively, with triplicate measurements ensuring statistical validity.

### 2.6. Fourier-Transform Infrared Spectroscopy (FTIR)

The chemical composition of DFO-MS@SAIB and its individual components was determined using a Fourier-transform infrared (FTIR) spectrometer (Thermo Scientific Nicolet iS5, Waltham, MA, USA) across a wavenumber range of 500–4000 cm^−1^.

### 2.7. In Vitro Release Analysis

DFO-MS@SAIB and DFO-MS were immersed in 1 mL of PBS (pH 7.4) and incubated in a thermostatic shaker (37 °C, 60 rpm). At predetermined intervals (1, 3, 5, 7, 14, 21, 28, and 35 days), 0.5 mL of supernatant was collected from each preparation and replaced with an equal volume of fresh PBS to maintain sink conditions. The harvested supernatant was reacted with excess iron (III) chloride (≥99% purity; Sigma-Aldrich, St. Louis, MO, USA), and the DFO concentration in both formulations was quantified at 485 nm using a UV-Vis spectrophotometer (Thermo Fisher Scientific), with triplicate measurements to ensure analytical precision.

### 2.8. Dynamic Shear Rheological Characterization

The viscosity profiles of SAIB and DFO-MS@SAIB were analyzed using a rotational rheometer (MCR 302, Anton Paar, Graz, Austria) equipped with a cone-plate geometry (25 mm diameter, 1° cone angle). Samples were loaded at 25 °C and subjected to three sequential test modes: time-dependent viscosity at a constant shear rate (10 s^−1^, 120 s), temperature-dependent viscosity (25–50 °C, 10 s^−1^ shear rate), and shear rate-dependent viscosity (0–100 s^−1^, 25 °C), with triplicate measurements to ensure reproducibility, and the results were expressed as the apparent viscosity (η, Pa·s) versus time, temperature, or shear rate.

### 2.9. Cytocompatibility and Proliferation Assessment

Primary human umbilical vein endothelial cells (HUVECs; Cellverse Bio, Shanghai, China) served as in vitro models to investigate the biocompatibility of DFO-MS and DFO-MS@SAIB and their effects on the proliferation of cells. The acute biocompatibility of DFO-MS (control, 3 mg, 5 mg) on HUVECs was evaluated via live/dead staining (Calcein/PI Live/Dead Viability/Cytotoxicity Assay Kit; Beyotime, Shanghai, China) after 24 h incubation to capture burst-release-induced cytotoxicity, whereas the sustained proliferative effects of DFO-MS@SAIB (Control, 6 mg, 8 mg, 10 mg, 12 mg) were assessed using CCK-8 assays (Dojindo, Kumamoto, Japan) over 7 days. Cells were cultured in 24-well plates (1 × 10⁴ cells/well), treated with designated formulations, and analyzed using fluorescence microscopy (Nikon Eclipse Ts2, Nikon, Tokyo, Japan; Live/Dead Viability Assay Corning, Corning, NY, USA) or microplate absorbance (450 nm; CCK-8), with triplicate experiments normalized to untreated controls.

### 2.10. In Vitro Angiogenic Evaluation of HUVECs

To evaluate the angiogenic efficacy of DFO-MS and DFO-MS@SAIB, a standardized endothelial tubulogenesis assay was performed using HUVECs cultured in Matrigel matrix (Corning, NY, USA). Prior to experimentation, cells were starved in basal medium (0.5% fetal bovine serum FBS ((Fetal Bovine Serum) Gibco, Grand Island, NY, USA) for 6 h to synchronize proliferation. A 24-well plate was coated with 20 μL Matrigel per well and polymerized at 37 °C for 30 min. HUVECs (1.5 × 10⁴ cells/well) were seeded onto the gel and immediately treated with the designated groups: DFO-MS were allocated into four groups, namely Control, 1 mg, 2 mg, and 3 mg (upper limit determined by CCK-8), while DFO-MS@SAIB comprised Control, 6 mg, 8 mg, and 10 mg (upper limit determined by CCK-8). Cells were incubated under standard conditions (37 °C, 5% CO_2_) for 6 h. Tubule networks were imaged using microscopy (Nikon, Tokyo, Japan).

### 2.11. Wound Healing and Transwell Migration Assays

After dose selection based on endothelial angiogenesis and viability, HUVECs pretreated with the respective doses were co-cultured with bone mesenchymal stem cells (BMSCs; Cellverse Bio, Shanghai, China) in a Transwell system (0.4 μm pore, Corning) for 48 h. The BMSCs were then harvested for dual-migration evaluation. For the wound-healing assay, BMSCs (2 × 10⁵ cells/well) were seeded into 6-well plates, grown to 90% confluence in Dulbecco’s Modified Eagle Medium (DMEM, Gibco)/10% FBS, and synchronized in 2% FBS medium for 12 h. Uniform scratches were created with a 200 μL pipette tip. Cells were allocated into four groups: Control (basal medium), DFO-MS, and DFO-MS@SAIB. Migration distances (μm) were quantified at 12/24 h. For the Transwell migration assays, BMSCs (1 × 10⁵ cells) were seeded into Transwell upper chambers (8 μm pore; Corning). The lower chambers contained conditioned media from the respective co-culture groups. After 12 h, migrated cells were fixed (4% paraformaldehyde), stained (0.1% crystal violet), and counted in five random fields/membrane.

### 2.12. Alkaline Phosphatase (ALP) and Alizarin Red Staining (ARS)

Osteogenic induction medium enriched with high-glucose DMEM containing 50 μg/mL ascorbic acid, 10 mM β-glycerophosphate, and 10 nM dexamethasone was prepared. As described in Section 2.11, BMSCs (1 × 10⁴ cells/well) were seeded in 24-well plates and divided into three groups: Control, DFO-MS, and DFO-MS@SAIB. BMSCs co-cultured with HUVECs were induced in osteogenic medium for 14 and 21 days. A colorimetric ALP assay kit (Beyotime, Shanghai, China) and 2% Alizarin Red S Staining Solution (Beyotime, China) was used to locate ALP-positive cells and mineralized nodules.

### 2.13. Fluorescence Immunostaining

BMSCs co-cultured with HUVECs (Control, DFO-MS, or DFO-MS@SAIB groups) were seeded onto glass coverslips in 24-well plates and osteogenically induced for 7 days. Cells were fixed with 4% paraformaldehyde (15 min, RT; Dowobio, Dalian, China), permeabilized with 0.1% Triton X-100 (10 min, RT; Dowobio, Dalian, China), and blocked in 5% BSA (1 h, RT; Solarbio, Beijing, China). A primary antibody against Runx2 (rabbit monoclonal, 1:200; Servicebio, Wuhan, China) was applied overnight at 4 °C, followed by incubation with an Alexa Fluor 488-conjugated goat anti-rabbit secondary antibody (2 h, RT, 1:500; Servicebio, Wuhan, China). Nuclei were counterstained with DAPI (5 μg/mL, 5 min; Servicebio, Beijing, China). Images were captured using a confocal microscope (Nikon A1R) with 40× objective. Runx2 fluorescence intensity was quantified via Image J (1.8.0.112).

### 2.14. Western Blotting

HUVECs were treated with different amounts of DFO-MS (1 mg, 2 mg, 3 mg) and DFO-MS@SAIB with different microsphere-loading amounts (6 mg, 8 mg, 10 mg), and BMSCs from the co-culture groups (Control, DFO-MS, DFO-MS@SAIB) were osteogenically induced for 14 days, lysed in RIPA buffer (containing protease/phosphatase inhibitors), and quantified via the BCA assay. Equal amounts of protein (30 μg/lane) were separated on 10% SDS-PAGE gels and transferred to PVDF membranes (Bio-Rad, Hercules, CA, USA). The membranes were blocked with 5% non-fat milk (1 h, RT) and incubated overnight (4 °C) with primary antibodies: anti-Hif-1α (rabbit monoclonal, 1:1000 in 5% BSA; Abcam, Cambridge, MA, USA), anti-Runx2 (rabbit monoclonal, 1:1000 in 5% BSA; Abcam), anti-Col1a1 (rabbit monoclonal, 1:1000 in 5% BSA; Abcam). After washing, the membranes were incubated with HRP-conjugated secondary antibodies (goat anti-rabbit, 1:5000; Abcam) for 1 h (RT). Bands were visualized using enhanced chemiluminescence (ECL; Advansta, San Jose, CA, USA), which were then analyzed using Image J (1.8.0.112).

### 2.15. In Vivo Bone Regeneration Experiments

A bilateral 5 mm critical-sized calvarial-defect model was established in male Sprague–Dawley rats (8 weeks, 300–350 g) under isoflurane anesthesia. Each rat served as its own internal control: the left defect was set as the Control group, while the right defect received either a DFO-MS or DFO-MS@SAIB (*n* = 5 per group) treatment, with doses corresponding to in-vitro-optimized concentrations (2 mg DFO-MS or 8 mg DFO-MS@SAIB per defect). Postoperative care included analgesia (buprenorphine) and antibiotics (cefazolin) for 3 days. At 8 weeks post-surgery, calvarial specimens were harvested, fixed in 4% paraformaldehyde, and subjected to micro-CT scanning (Scanco Medical, Bassersdorf, Switzerland) at 10 μm resolution for 3D reconstruction and quantification of bone morphometric parameters, including bone volume fraction (BV/TV) and bone mineral density (BMD).

Following micro-CT analysis, the samples were decalcified in 14% EDTA for 4 weeks, paraffin-embedded, and sectioned sagittally (5 μm thickness). Serial sections underwent H&E staining to evaluate the defect bridging and general tissue architecture and Masson’s trichrome staining to visualize collagen deposition (blue) and the osteoid matrix (red/pink). For immunohistochemical (IHC) analysis, antigen retrieval was performed in citrate buffer (pH 6.0), followed by blocking of endogenous peroxidase. Sections were incubated overnight with primary antibodies against Runx2 (1:200; Abcam) or Col1a1 (1:300; Abcam), then developed using HRP-conjugated secondary antibodies and the DAB chromogen. Nuclei were counterstained with hematoxylin.

Image quantification was performed using ImageJ: Masson’s trichrome for the new-bone-area fraction and IHC slides for the Runx2/Col1a1-positive signal intensity (integrated optical density normalized to tissue area). For statistical comparisons, paired *t*-tests were applied to evaluate differences between each experimental defect (DFO-MS or DFO-MS@SAIB) and its contralateral control within the same animal, with significance defined as *p* < 0.05 (GraphPad Prism 9.0).

## 3. Results and Discussion

### 3.1. Characterization of DFO-MS and DFO-MS@SAIB

**Figure 1:**
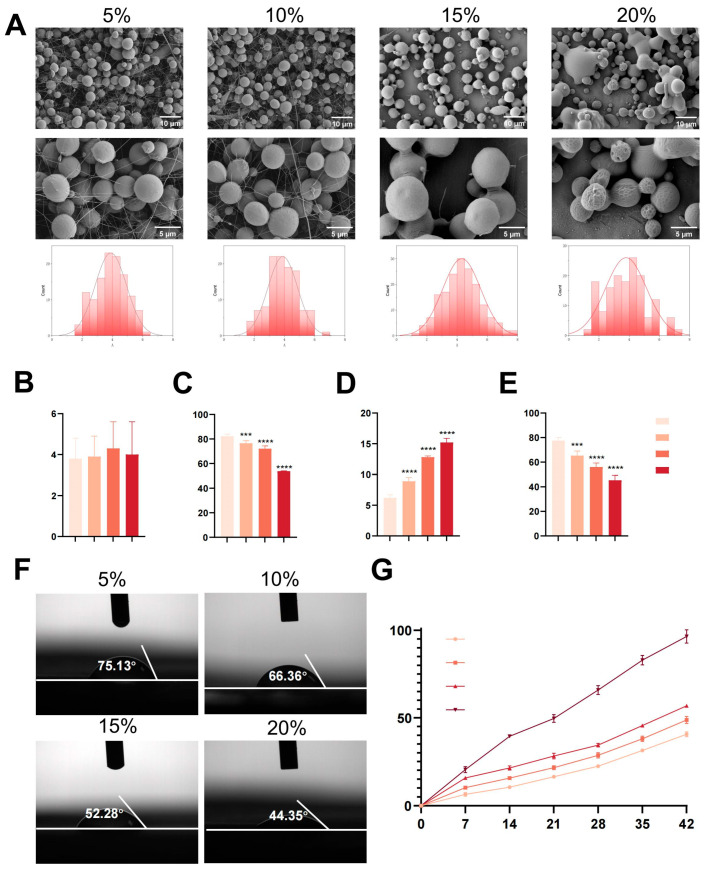
Characterization of the DFO-MS. (**A**) SEM images of microspheres with different drug-loading rates and their respective particle size distributions. (**B**–**E**) The statistical analysis of four indicators related to particle size, EE, EE, and contact angle for DFO-MS with different drug-loading rates. (**F**) Contact-angle measurements of DFO-MS with different drug-loading rates. (**G**) In vitro degradation of different microspheres. Error bars, mean ± standard deviation; *** *p* < 0.001; **** *p* < 0.0001 (*n* = 5).

DFO-MS with drug loading (DL) rates of 5%, 10%, 15%, and 20% were fabricated using an electrostatic spray technique. Scanning electron microscopy (SEM) analysis revealed that microspheres with a DL ≤ 15% predominantly exhibited a spherical morphology with smooth surfaces and near-normal size distributions (3.8–4.3 μm), although sporadic fibrous polymer skeletons were observed (Figure 1A). In contrast, microspheres with a DL > 15% displayed significant fusion, collapse, and polydisperse sizing, while DL = 15% microspheres achieved a uniform morphology with minimal fibrous structures. Size quantification confirmed no significant differences in mean diameter across the groups (*p* > 0.05, one-way ANOVA; Figure 1B). The inherent incompatibility between hydrophilic DFO and hydrophobic PLGA led to drug–polymer phase separation during fabrication, consistent with prior reports of hydrophilic drug-induced surface pitting in PLGA matrices [35]. This phenomenon may account for the abnormal morphological features observed in microspheres with 20% DL. Furthermore, drug-loaded microspheres can develop structural anomalies such as filamentous fibers, which are potentially attributable to either insufficient compatibility between the therapeutic agent and the polymeric matrix or heterogeneous solution concentration distributions resulting from suboptimal DLs [36,37] Contact-angle measurements demonstrated enhanced hydrophilicity with increasing DL (77.5° ± 2.5 for 5% DL vs. 45.4° ± 3.9 for 20% DL; Figure 1E,F). Notably, DFO-MS displayed lower contact angles than pure PLGA films, with values decreasing from 77.5° ± 2.5° (5% DFO) to 45.4° ± 3.9° (20% DFO), reflecting the hydrophilic effect of DFO on the microsphere surface, which promoted interparticle fusion due to reduced interfacial stability. While increasing the DFO-to-PLGA ratio elevated the drug loading efficiency (LE) from 6.2% ± 0.5 (5% DL) to 15.2% ± 0.7 (20% DL), the encapsulation efficiency (EE) decreased from 82.2% ± 1.7 to 53.8% ± 0.2, which was attributed to rapid DFO diffusion from the organic phase to the aqueous phase during emulsification [38]. In vitro degradation studies (Figure 1G) revealed that 15% DL microspheres retained ~70% of their mass after 4 weeks in PBS, whereas high-DL groups (≥20%) exhibited accelerated mass loss (>50%) driven by polymer-chain relaxation and DFO-induced hydration via hydrophilic drug–water interactions [6,7]. These findings confirm that a 15% DL serves as a critical threshold, balancing microsphere morphology, structural stability, and drug retention.

### 3.2. Characterization of DFO-MS@SAIB

**Figure 2:**
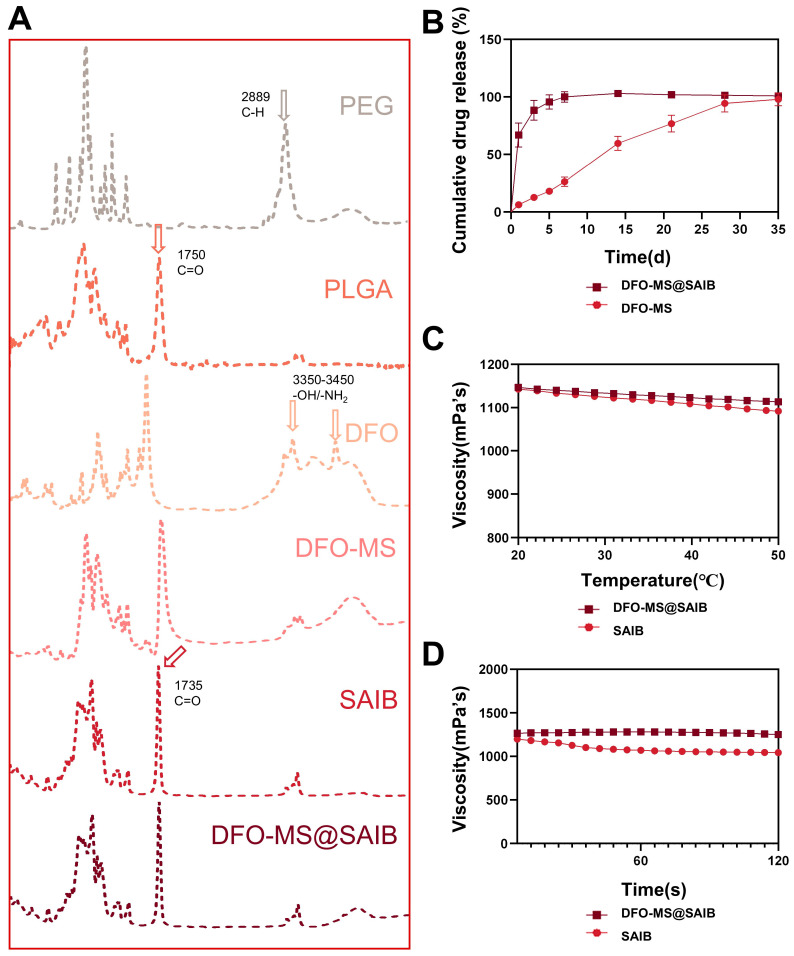
Characterization of DFO-MS and DFO-MS@SAIB. (**A**) FTIR spectroscopy analysis of DFO-MS and DFO-MS@SAIB (the arrows in the figure denote the characteristic peak wavelengths and associated chemical bonds). (**B**) Cumulative drug release of DFO-MS and DFO-MS@SAIB. (**C**) The viscosity of SAIB and DFO-MS@SAIB at varying temperatures (°C). (**D**) The viscosity changes in SAIB and DFO-MS@SAIB over time(s).

FTIR spectroscopy revealed characteristic vibrations: DFO’s -OH/-NH_2_ at 3350–3450 cm^−1^, PLGA’s ester C=O at 1750 cm^−1^, SAIB’s ester C=O at 1735 cm^−1^, and PEG’s C-H at 2889 cm^−1^, confirming the physical blending of components. FTIR spectral analysis (Figure 2A) identified SAIB’s strong ester C=O stretching vibration at 1735 cm^−1^. DFO displayed a broad absorption band at 3350–3450 cm^−1^, attributed to overlapping -OH and -NH_2_ stretching vibrations, while PLGA exhibited characteristic peaks at 1750 cm^−1^ (ester C=O) and 2980 cm^−1^ (C-H stretching of -CH_3_/-CH_2_-). PEG signatures included C–H stretching (2889 cm^−1^) and bending (1470 cm^−1^). The DFO-MS spectrum retained all constituent peaks (DFO’s -OH at 3400 cm^−1^, PLGA’s C=O at 1750 cm^−1^, PEG’s C-H at 2889 cm^−1^) without peak shifts or new bonds, confirming physical blending without covalent interactions. Comparative analysis of DFO-MS@SAIB and SAIB spectra revealed high consistency across most wavelengths, with notable divergence at 3000–3500 cm^−1^. DFO-MS@SAIB introduced an additional broad -OH stretching band (~3400 cm^−1^) from DFO’s polyhydroxy structure and intermolecular hydrogen bonding, which is distinguishable from PEG’s C-H peaks by its peak shape and position.

The SAIB-based formulation comprises a hydrophobic solvent, a therapeutic agent (DFO), and release-modulating excipients, with the viscosity adjusted via minimal organic solvents for injectability. Upon in vivo injection, solvent exchange with tissue fluids triggers SAIB self-assembly into a high-viscosity depot for sustained drug release [39]. Conventional SAIB systems exhibit delayed depot formation, leading to an initial burst release due to drug–solvent diffusion, whereas integration with microspheres (DFO-MS@SAIB) suppresses this burst effect, reducing the initial release by an order of magnitude [10]. In vitro drug-release profiles (Figure 2B) demonstrated rapid DFO release from DFO-MS (60% within 24 h) versus sustained release from DFO-MS@SAIB (75% cumulative release over 7 days). The rheological analysis revealed shear-thinning behavior in both SAIB and DFO-MS@SAIB, but the latter exhibited optimized viscoelastic properties: the viscosity at 25 °C and 100 s^−1^ (simulating injection) increased significantly compared with SAIB (Figure 2D), while the temperature sensitivity decreased (viscosity at 37 °C: 1000 mPa·s, 2.9% reduction vs. 4.4% for SAIB), ensuring injectability (Figure 2C). The DFO-MS@SAIB formulation exhibited markedly elevated viscosity at 25 °C/100 s^−1^ compared with SAIB, a rheological property that enables maintenance of a stable fluidic state during needle administration, thereby mitigating risks of either drug leakage from insufficient viscosity or needle occlusion. Concurrently, its attenuated thermoresponsiveness (demonstrated by only a 2.9% viscosity reduction at 37 °C versus 4.4% in SAIB) ensures a more gradual viscoelastic transition during an environmental temperature shift from ambient to physiological conditions, ultimately guaranteeing precise therapeutic delivery to target sites.

This integrated physicochemical profiling validates the structural integrity and sustained-release mechanism of DFO-MS@SAIB, aligning with injectable-depot design principles for controlled drug delivery.

### 3.3. Dose Selection Based on Endothelial Angiogenesis and Viability

**Figure 3:**
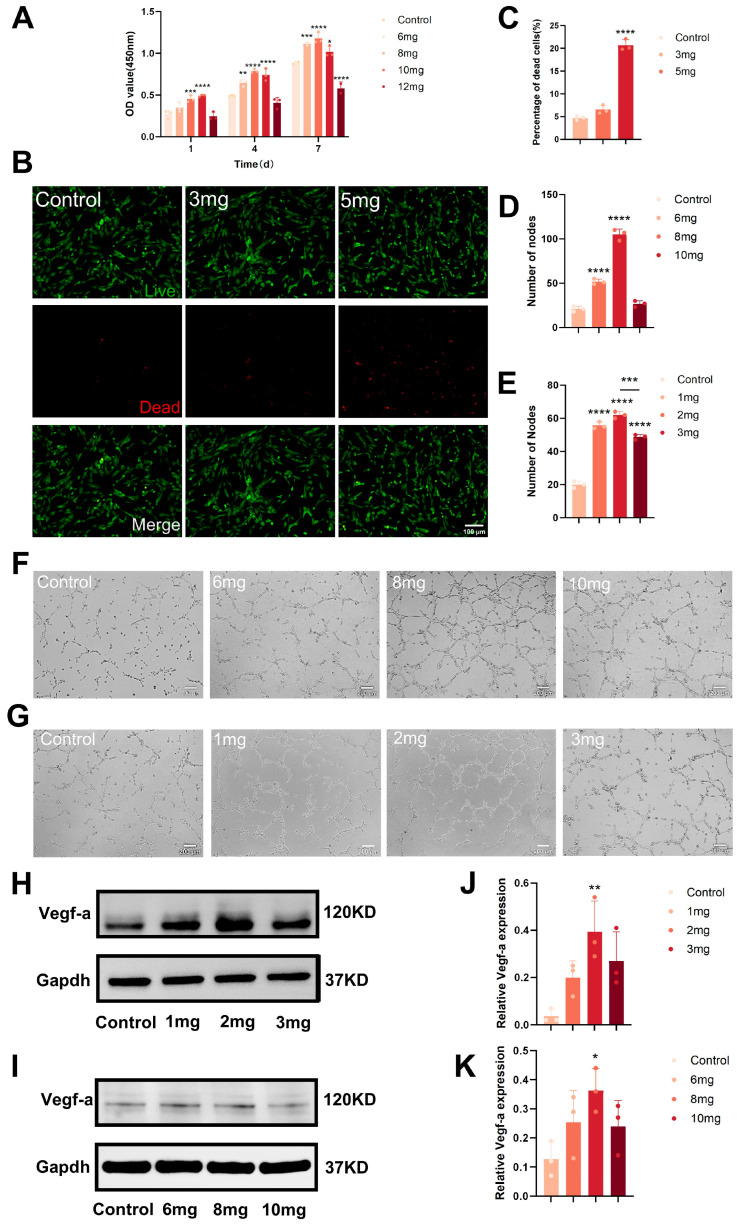
Dose selection based on endothelial angiogenesis and viability in osteogenic co-culture. (**A**) Quantitative analysis of cell proliferation of HUVECs treated by DFO-MS@SAIB with different microsphere-loading amounts. (**B**) Live/Dead-staining of HUEVCs cultured with different amounts of DFO-MS. (**C**) The dead HUVEC rate under PI labeling. (**D**,**E**) Quantitative analysis by the number of nodes. (**F**) Tube formation of HUVECs treated by DFO-MS@SAIB with different microsphere-loading amounts. (**G**) Tube formation of HUVECs treated with different amounts of DFO-MS. (**H**,**I**) Expression of the VEGF-a protein in HUEVCs treated with different amounts of DFO-MS and DFO-MS@SAIB with different microsphere-loading amounts. (**J**,**K**) Quantitative analysis of the relative VEGF-a expression. Error bars, mean ± standard deviation; * *p* < 0.05; ** *p* < 0.01; *** *p* < 0.001; **** *p* < 0.0001 (*n* = 3).

Dose screening for DFO-MS and DFO-MS@SAIB in osteogenic co-culture systems prioritized balancing HUVEC angiogenic functionality with viability to maximize material efficacy. For the sustained-release DFO-MS@SAIB, a 7-day CCK-8 assay confirmed no significant cytotoxicity at microsphere loadings of 1–10 mg within the SAIB matrix compared to the control group, validating its long-term biocompatibility (Figure 3A). In contrast, the burst-release DFO-MS required acute toxicity screening via live/dead staining due to risks of transient local drug overload. The cell mortality remained at <10% at direct microsphere doses of 1–3 mg, establishing a preliminary safe range (Figure 3B–E). Within these non-cytotoxic ranges, endothelial tubulogenesis assays revealed that DFO-MS@SAIB with an 8 mg microsphere loading demonstrated superior pro-angiogenic efficacy, with vascular node counts (105 ± 6.2) significantly exceeding both those of the control (21 ± 3.1, *p* < 0.01) and the optimal DFO-MS dose of 2 mg (62 ± 2.0), which can be attributed to its sustained drug-release profile (Figure 3F,G).

Western blot analysis showed dose-dependent modulation of VEGF-a expression. In DFO-MS-treated groups (Figure 3H,J), 1 mg and 2 mg microsphere doses upregulated VEGF-a versus the control, suggesting low-dose stabilization of VEGF-a, while the 3 mg dose induced downregulation, likely due to cellular stress. For DFO-MS@SAIB (Figure 3I,K), the 6 mg and 8 mg microsphere loadings robustly enhanced VEGF-a expression, whereas the 10 mg loading caused abrupt suppression. Both systems exhibited a “low-promotion, high-inhibition” pattern, underscoring the necessity for precise dose-loading optimization (e.g., 2 mg dose for DFO-MS; 8 mg loading for DFO-MS@SAIB) to activate VEGF-a-mediated pro-angiogenic signaling while avoiding toxicity. Collectively, 2 mg DFO-MS and 8 mg DFO-MS@SAIB represent the optimal dosing ranges, balancing pro-angiogenic activity (e.g., tubulogenesis node count) and cytocompatibility (viability > 90%) in co-culture systems.

### 3.4. In Vitro Osteogenesis of BMSCs in Endothelial Co-Culture Systems

**Figure 4:**
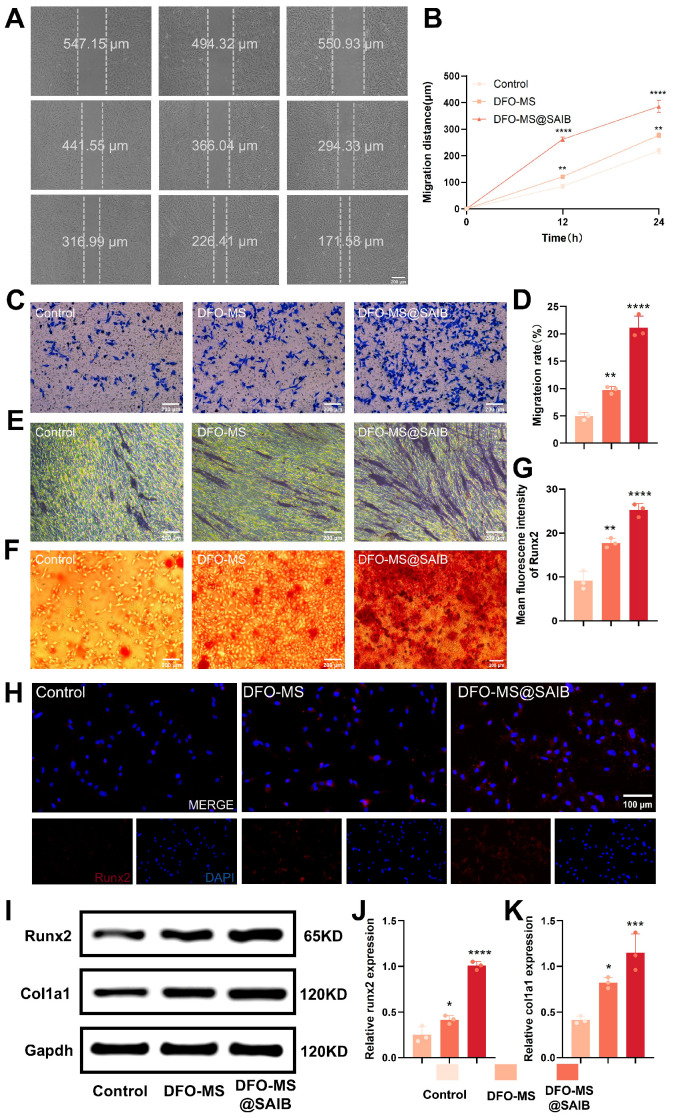
Osteogenic functional analysis of BMSCs in an endothelial co-culture system. (**A**) Wound-healing process of BMSCs and semiquantitative analysis (wound width). (**B**) Quantitative analysis of the wound-healing process at 12 h and 24 h. (**C**) Migration ability of BMSCs evaluated by the Transwell system. (**D**) Quantitative analysis of the migrated BMSCs. (**E**) ALP staining of BMSCs at day 7. (**F**) ARS staining of BMSCs at day 21. (**G**) Quantitative analysis of Runx2 fluorescence. (**H**) Representative Runx2 fluorescence image of BMSCs. (**I**) Expression of proteins Runx2 and Col1a1 in BMSCs. (**J**,**K**) Quantitative analysis of the relative Runx2 and Col1a1 expression. Error bars, mean ± standard deviation; * *p* < 0.05; ** *p* < 0.01; *** *p* < 0.001; **** *p* < 0.0001 (*n* = 3).

The osteogenic functionality of BMSCs in endothelial co-culture systems revealed a significant enhancement mediated by DFO-MS@SAIB. Scratch wound-healing assays (Figure 4A,B) demonstrated that BMSCs treated with DFO-MS@SAIB exhibited markedly reduced wound widths over time, with migration distances at 24 h substantially exceeding those of the control and DFO-MS groups, indicating superior migratory capacity. Transwell migration assays (Figure 4C,D) further confirmed this trend, showing significantly higher BMSC migration rates in the DFO-MS@SAIB group compared with all others (*p* < 0.0001), underscoring its efficacy in promoting cellular recruitment to injury sites—a critical factor in bone-defect repair.

The osteogenic differentiation assays highlighted robust pro-osteogenic effects. At day 7, ALP staining (Figure 4E) revealed intensified alkaline phosphatase activity in the DFO-MS@SAIB group, while Alizarin Red S (ARS) staining at day 21 (Figure 4F) demonstrated a substantial increase in calcium nodule formation, indicative of advanced mineralization. Immunofluorescence and Western blot analyses further elucidated mechanistic insights: Runx2, a master transcription factor for osteogenesis, exhibited significantly higher fluorescence intensity (Figure 4G,H) and Runx2 expression (Figure 4I,J) in the DFO-MS@SAIB group versus the controls (*p* < 0.0001). Similarly, Col1a1 expression (Figure 4I,K) was markedly upregulated (*p* < 0.001), corroborating enhanced extracellular matrix synthesis. The co-culture system revealed that sustained DFO release from DFO-MS@SAIB enhances endothelial cell (HUVEC) functionality in the co-culture system, thereby activating osteogenic differentiation pathways in BMSCs through paracrine crosstalk. The enhanced angiogenic activity of HUVECs—evidenced by increased vascular node formation—triggers the secretion of pro-osteogenic mediators such as VEGF, BMP-2, and PDGF. These factors drive BMSC migration and lineage commitment to osteoblasts. Mechanistically, DFO chelates iron ions to inhibit PHD activity, blocking the hydroxylation and ubiquitination-mediated degradation of HIF-1α [40]. Stabilized HIF-1α translocates to the nucleus, forms a transcription complex with HIF-1β, and activates downstream target genes (e.g., VEGF, BMP-2) [41,42,43]. This endothelial-to-mesenchymal signaling axis explains the upregulation of Runx2 and Col1a1 in BMSCs, where endothelial-derived factors synergize with sustained DFO signaling to amplify osteogenic marker expression. This effect is mediated by DFO activation of the HIF-1α/VEGF pathway [44]: VEGF binds to VEGFR2 on endothelial cells [45], activating the downstream PI3K-AKT signaling pathway to promote osteoblastic differentiation and cytoskeletal reorganization in BMSCs [46].

The osteogenic functionality of BMSCs in an endothelial co-culture system was significantly enhanced by DFO-MS@SAIB treatment. This highlights the importance of endothelial cells in regulating the osteogenic differentiation of mesenchymal stem cells through paracrine signaling. The DFO-MS@SAIB system facilitates this process by enhancing both endothelial and osteogenic functions, which ultimately leads to improved bone healing and regeneration [47]. Future studies could explore the clinical applicability of this approach in vivo, particularly in models of bone defects, to validate the translational potential of DFO-MS@SAIB for enhancing bone repair. Additionally, the precise molecular pathways involved in the endothelial-to-mesenchymal signaling axis could be further elucidated to refine the design of targeted therapeutics for bone tissue engineering.

### 3.5. In Vivo Osteogenesis in Calvarial Defects

**Figure 5:**
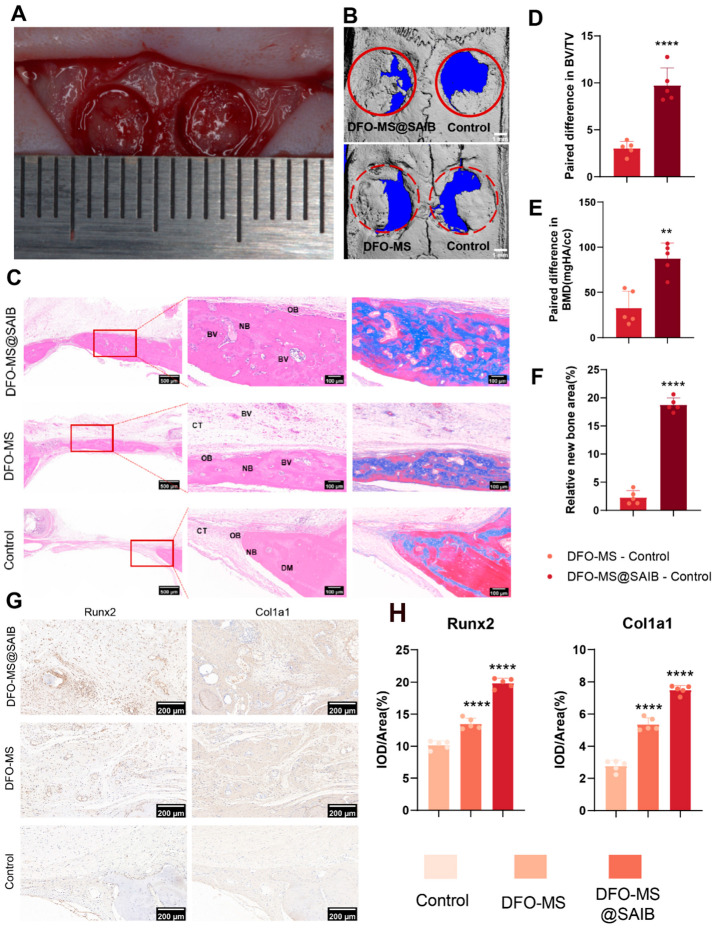
Bone regeneration efficacy in calvarial defects. (**A**) Establishment of a rat calvarial-defect model (defect size = 5 mm). (**B**) Rat skull 3D rendering at 8 weeks. (**C**) H&E staining and Masson‘s trichrome staining at 8 weeks (NB: new bone; DM: defect margin; OB: osteoblast; CT: connective tissue; BV: blood vessels). (**D**) Paired difference in BV/TV. (**E**) Paired difference in relative new-bone area. (**F**) Paired difference in BMD. (**G**) Immunohistochemical staining of Runx2 and Col1a1. (**H**) Quantitative evaluation of the expression of Runx2 and Col1a1. Error bars, mean ± standard deviation; ** *p* < 0.01; **** *p* < 0.0001 (*n* = 5).

In a critical-sized (5 mm) rat calvarial-defect model (Figure 5A), DFO-MS@SAIB demonstrated superior bone regenerative capacity. Micro-CT 3D reconstruction (Figure 5B) at 8 weeks post-implantation revealed significantly greater new bone formation in the DFO-MS@SAIB group compared with controls and DFO-MS, with defect bridging and trabecular network maturation visually evident. Histological analysis (Figure 5C) further corroborated these findings: DFO-MS@SAIB-treated defects exhibited dense connective tissue (CT) interspersed with robust neobone (NB), abundant blood vessels (BV), and active osteoblasts (OB), indicative of vascularized osteogenesis. In contrast, DFO-MS and control groups showed sparse NB with limited vascular infiltration, highlighting DFO-MS@SAIB’s unique ability to orchestrate a vascular-rich osteogenic microenvironment.

Quantitative morphometrics reinforced these observations: DFO-MS@SAIB achieved significantly higher paired differences in bone volume/total volume (BV/TV) (Figure 5D, *p* < 0.001) and relative new-bone area (Figure 5E, *p* < 0.01) versus the contralateral controls, underscoring its dual efficacy in enhancing bone volume and regeneration kinetics. Immunohistochemical profiling (Figure 5F–H) revealed intense Runx2 and Col1a1 expression in DFO-MS@SAIB-treated defects (*p* < 0.0001 vs. other groups), confirming its potent activation of osteogenic differentiation pathways.

These results highlight the multifunctional capabilities of DFO-MS@SAIB as a scaffold material for bone regeneration. Its ability to promote both mineralization and vascularized osteogenesis positions it as a promising candidate for clinical applications in bone-defect repair, particularly in scenarios that demand expedited healing and functional integration of the bone. Future studies could explore optimizing the scaffold’s properties further, such as its mechanical strength, biodegradability, and long-term healing outcomes, as well as its performance in other models of bone regeneration.

### 3.6. RNA-Seq Analysis of BMSCs and HUVECs Treated with DFO-MS@S

**Figure 6:**
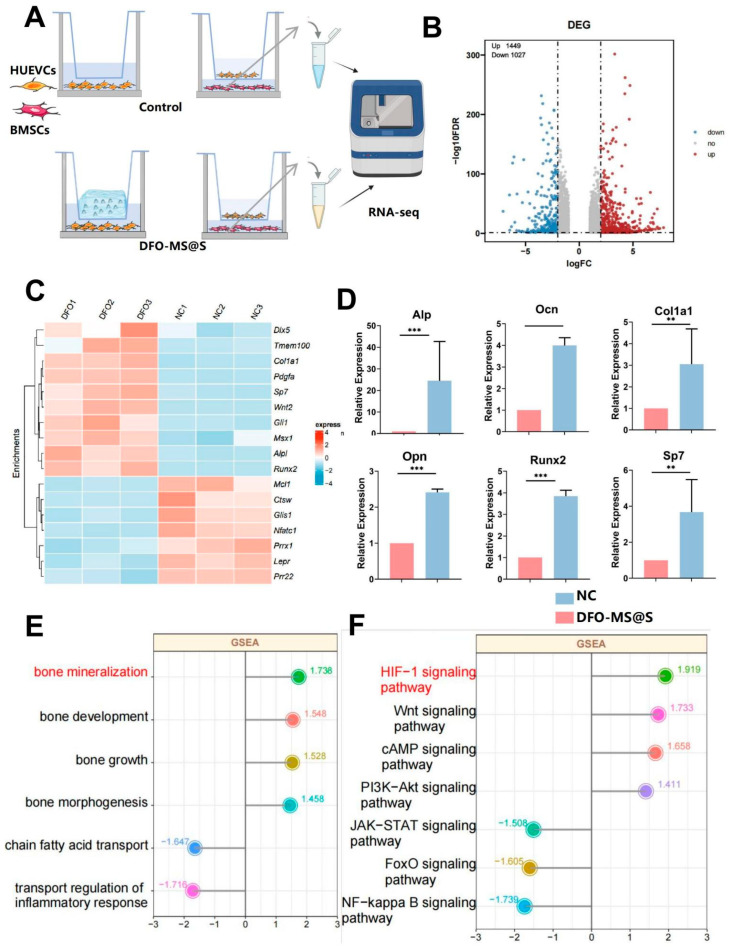
RNA-seq of DFO-MS@S effects on BMSCs. (**A**) RNA-seq reveals the underlying mechanisms of DFO-MS@S in BMSCs. (**B**) Volcano plot showing differentially expressed genes between the NC and DFO-MS@S groups. (**C**) Heatmap of osteogenesis-related gene expression between the NC and DFO-MS@S groups. (**D**) Bar plot of osteogenesis-related gene expression levels. (**E**,**F**) Enrichment analysis of GO functions and KEGG signaling pathways based on GSEA. Data are presented as the mean ± standard deviation. ** *p* < 0.01, *** *p* < 0.001 (*n* = 6).

The entire transcriptome of BMSCs co-cultured with HUVECs, treated with or without DFO-MS@S, was sequenced to investigate the mechanisms facilitating osteogenesis (Figure 6A). Differential expression gene (DEG) analysis was performed with a threshold of |log2 FC| ≥ 0 and a padj of ≤0.05. The volcano plot revealed that the DFO-MS@S-treated group had 1449 significantly upregulated genes and 1027 significantly downregulated genes (Figure 6B). Classical osteogenesis-related genes, such as Alpl, Runx2, and Col1a1, were highlighted in the heatmap, showing that these genes were upregulated in the DFO-MS@S-treated groups (Figure 6C). The bar plot further indicated that genes like Alp, Ocn, Col1a1, Opn, Runx2, and Sp7 were significantly upregulated after DFO-MS@S treatment (*p* < 0.01 vs. other groups) (Figure 6D). Gene-set enrichment analysis (GSEA) of GO and KEGG pathways confirmed that the differentially expressed genes (DEGs) were enriched in functions such as bone mineralization and signaling pathways like the HIF-1 signaling pathway (Figure 6E,F). Thus, we infer that DFO-MS@S facilitates bone mineralization through the HIF-1 signaling pathway.

In conclusion, this study elucidates that DFO-MS@S promotes osteogenesis in BMSCs by modulating gene expression related to osteoblast differentiation and bone mineralization, particularly through the HIF-1 signaling pathway [48]. These findings provide a deeper understanding of the molecular mechanisms underlying the osteogenic effects of DFO-MS@S, positioning it as a promising therapeutic strategy for bone tissue engineering. Future studies could explore the detailed roles of specific upregulated genes in osteogenesis and investigate the potential of DFO-MS@S in in vivo models to validate its clinical applications in bone regeneration.

## 4. Conclusions

This study developed DFO-loaded PLGA microspheres (DFO-MS) via electrospraying and combined them with SAIB to form an injectable depot (DFO-MS@SAIB) for bone-defect repair. Physicochemical properties (morphology, EE, degradation, and release kinetics) were characterized, verifying its dual roles in promoting angiogenesis and osteogenic differentiation. In vitro, DFO-MS@SAIB enhanced HUVEC angiogenic capacity and BMSC migration/differentiation via stabilizing HIF-1α and upregulating Runx2 and Col1a1. In a rat calvarial-defect model, DFO-MS@SAIB showed higher new bone formation (BV/TV) and vascularization than controls, confirming that sustained DFO release activates HIF-1α signaling for vascularized regeneration. This study provides a biocompatible, sustained-release strategy for critical bone defects with clinical translation potential.

## Data Availability

Data are contained within the article.
